# Supplementary material for: Design, Synthesis and Biological Evaluation of α-Synuclein Proteolysis-Targeting Chimeras
Source: Molecules. 2023 May 31;28(11):4458. doi: 10.3390/molecules28114458 (PMC10254247; doi:10.3390/molecules28114458)
Supplement: Supplementary file 1 [file molecules-28-04458-s001.zip › molecules-2368888-supplementary.pdf]

# Supplementary Information

## Design, Synthesis and Biological Evaluation of $\alpha$-synuclein Proteolysis-Targeting Chimeras

Tianzhi Wen, Jian Chen, Wenqian Zhang and Jiyan Pang *

*School of Chemistry, Sun Yat-Sen University, Guangzhou, 510006, P. R. China*

* Author to whom correspondence should be addressed; Jiyan Pang, e-mail: cespjy@mail.sysu.edu.cn; Tel.: +86-186-8887-0045.

## CONTENTS

**Figure S1-S9** $^{1}$H NMR spectra of compound **1-9** 1-5

**Figure S10-S18** $^{13}$C NMR spectra of compound **1-9** 6-10

**Figure S19-S27** HRMS spectra of compound **1-9** 11-15

**Figure S28** Referential Western Blot image of wild type, SNCA over-expressed, and PFF transfected SNCA over-expressed H293T Cell lines 16

**Figure S29** Compounds induced degradation of α-synuclein aggregates 17

**Figure S30** The cell viability data 18

**Table S1** The predicted log*P* values of synthesized compounds 19

Figure S1. $^{1}$H spectrum of compound **1** (400 MHz, chloroform-d)

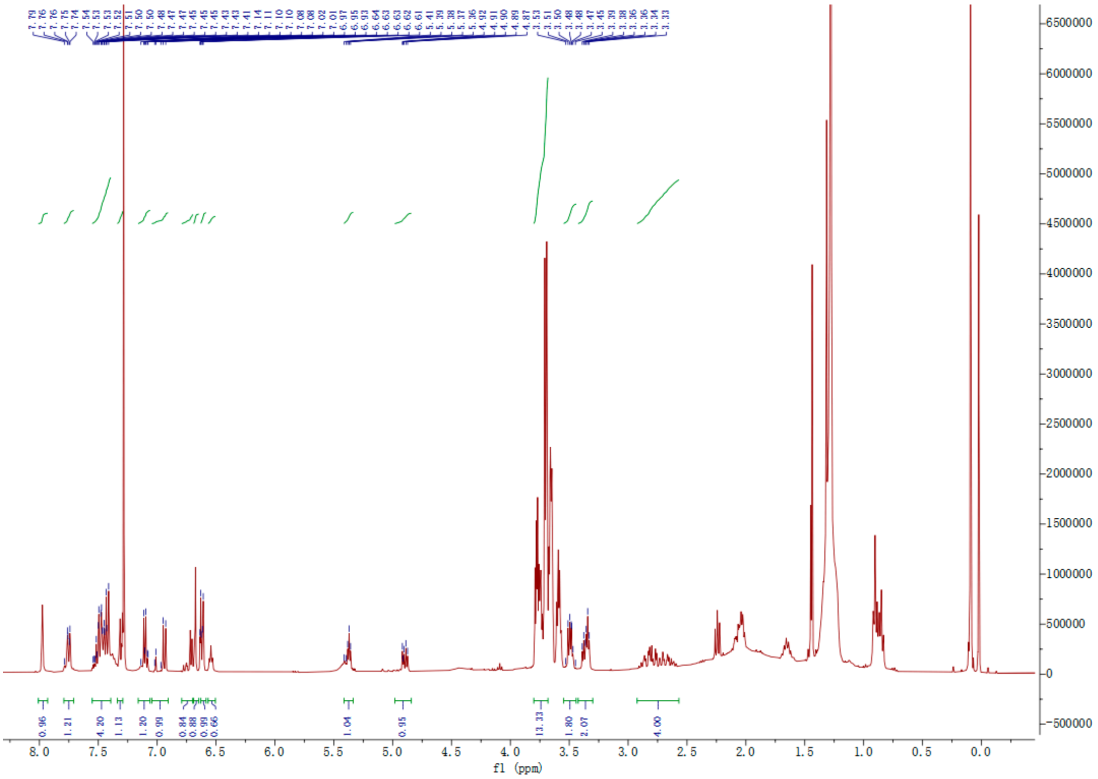

Figure S2. $^{1}$H spectrum of compound **2** (400 MHz, chloroform-d)

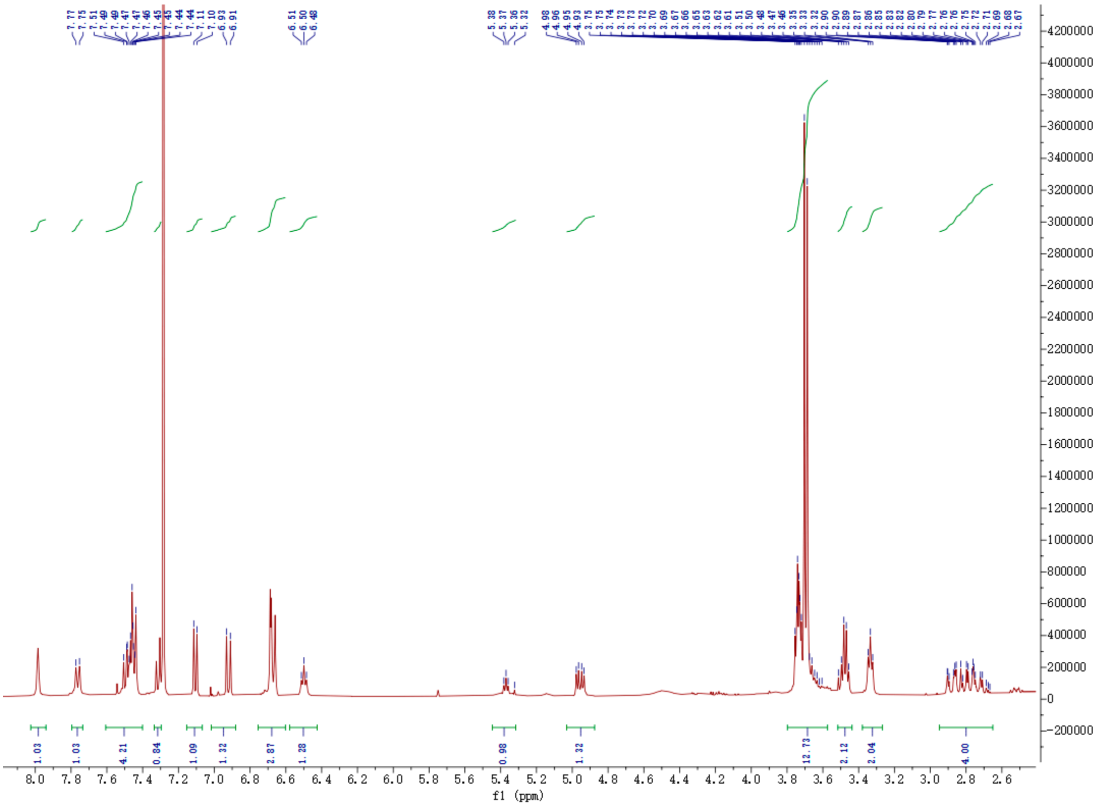

Figure S3. $^{1}$H spectrum of compound **3** (400 MHz, chloroform-d)

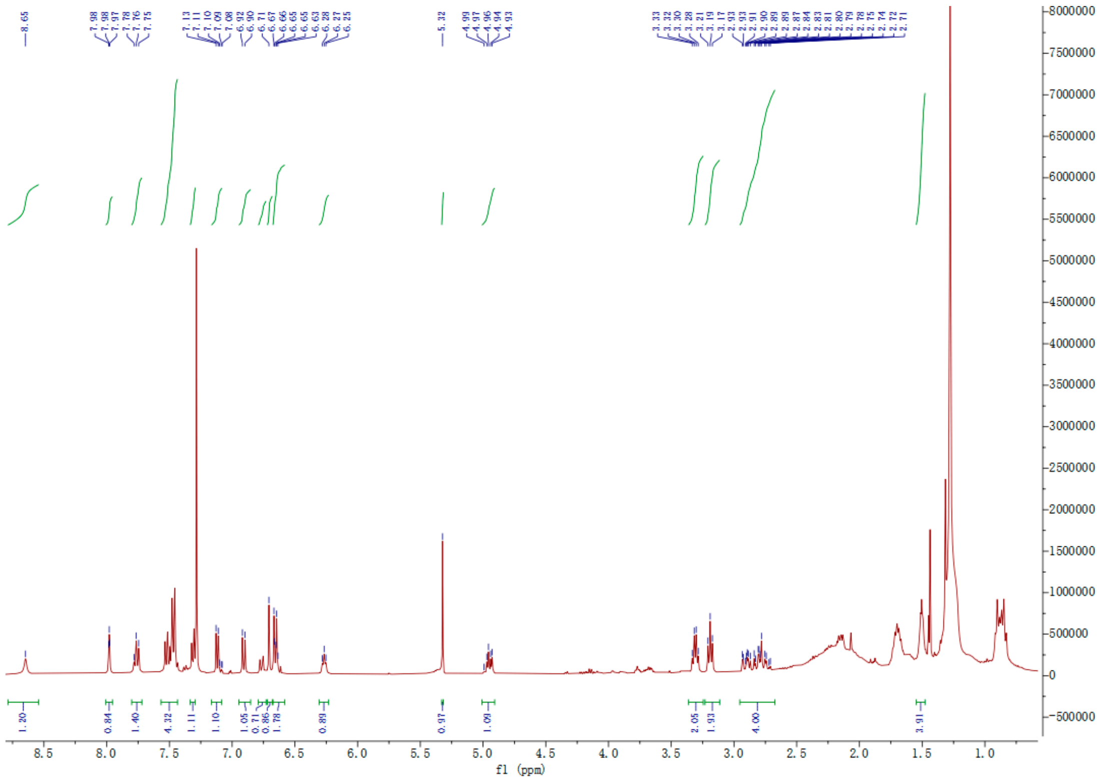

Figure S4. $^{1}$H spectrum of compound **4** (400 MHz, methanol-d4)

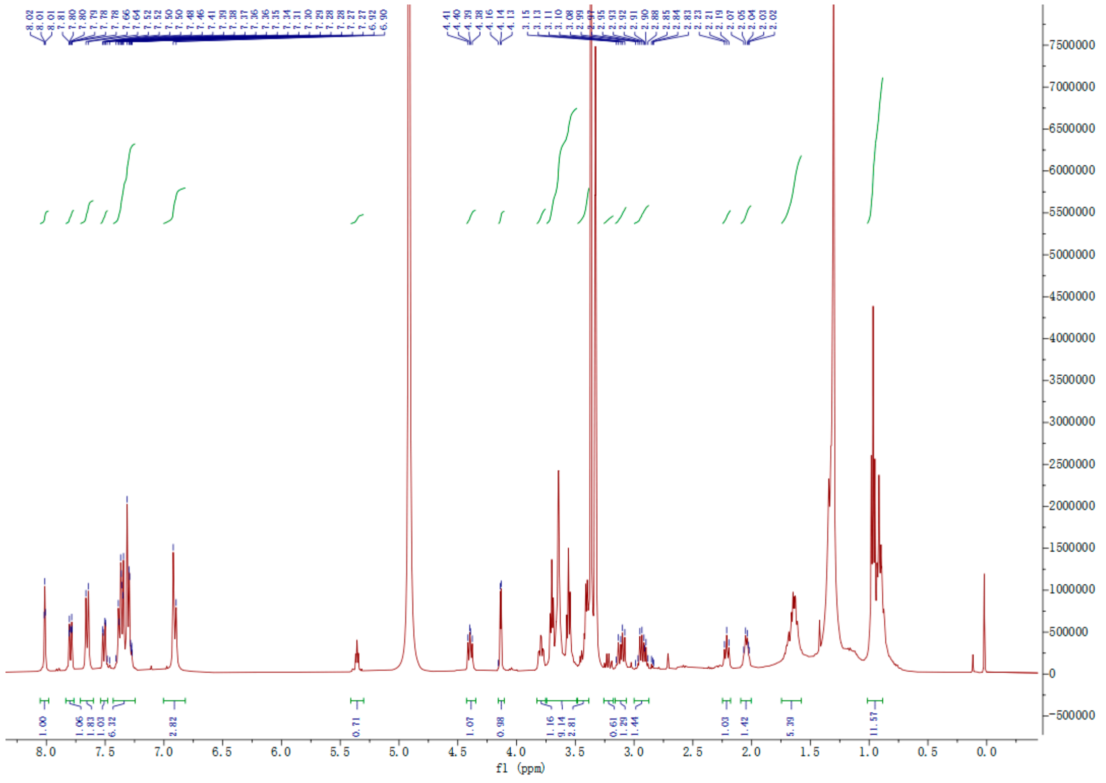

Figure S5. $^{1}$H spectrum of compound **5** (400 MHz, methanol-d4)

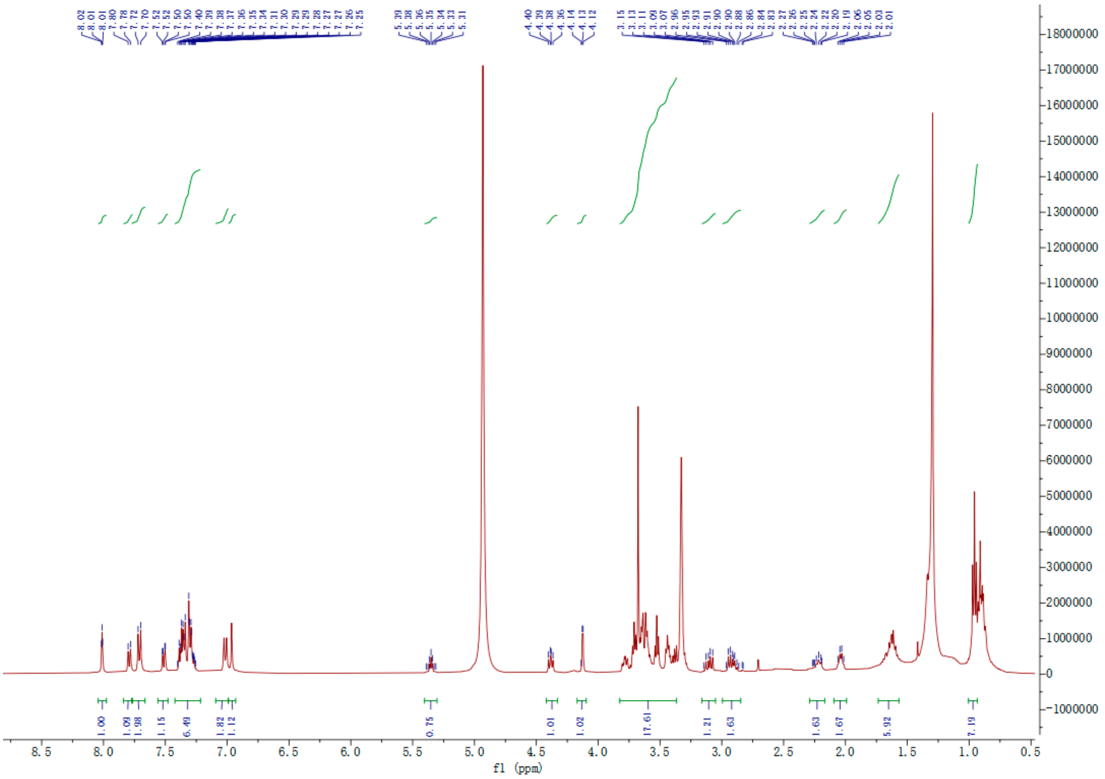


Figure S6. $^{1}$H spectrum of compound **6** (400 MHz, methanol-d4)

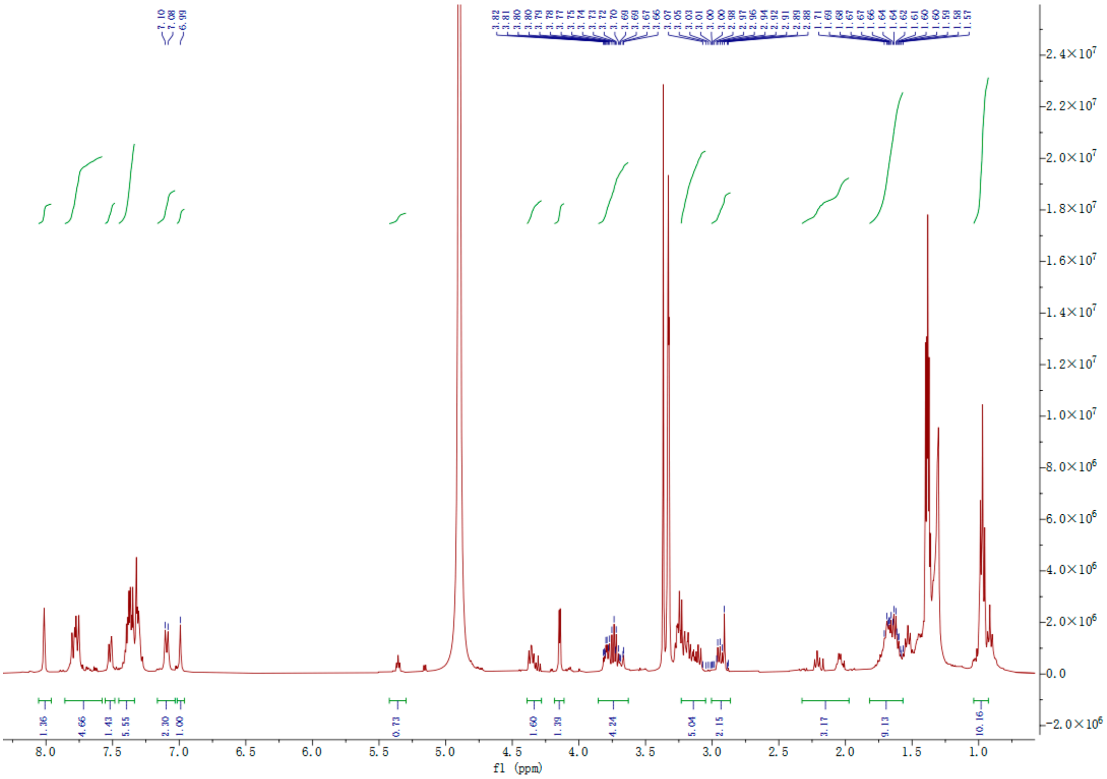

Figure S7. $^{1}$H spectrum of compound **7** (400 MHz, Methanol-d4)

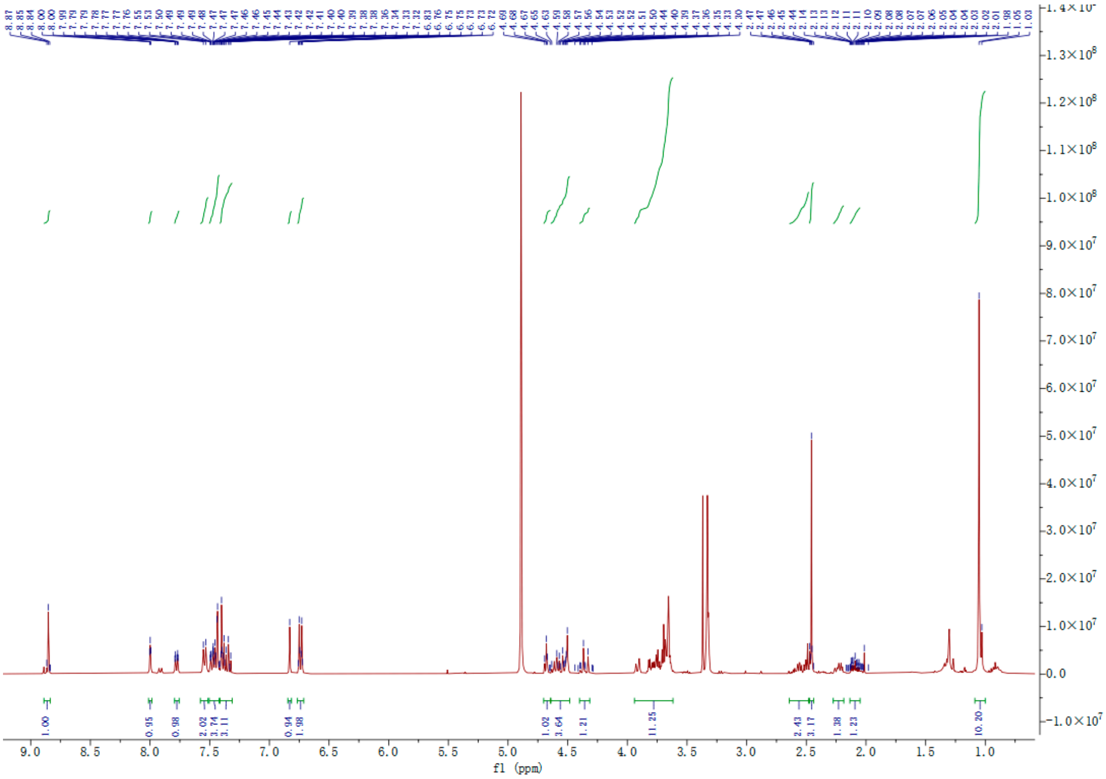

Figure S8. $^{1}$H spectrum of compound **36** (400 MHz, chloroform-d)

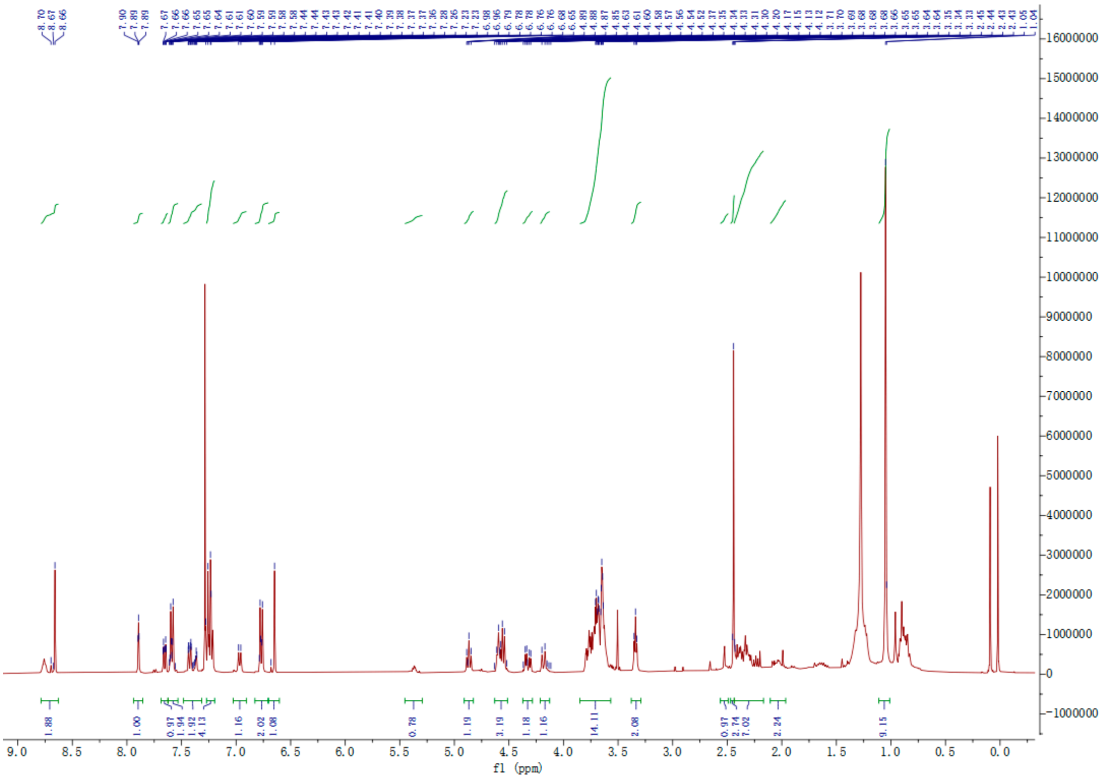

Figure S9. $^{1}$H spectrum of compound **9** (600 MHz, chloroform-d)

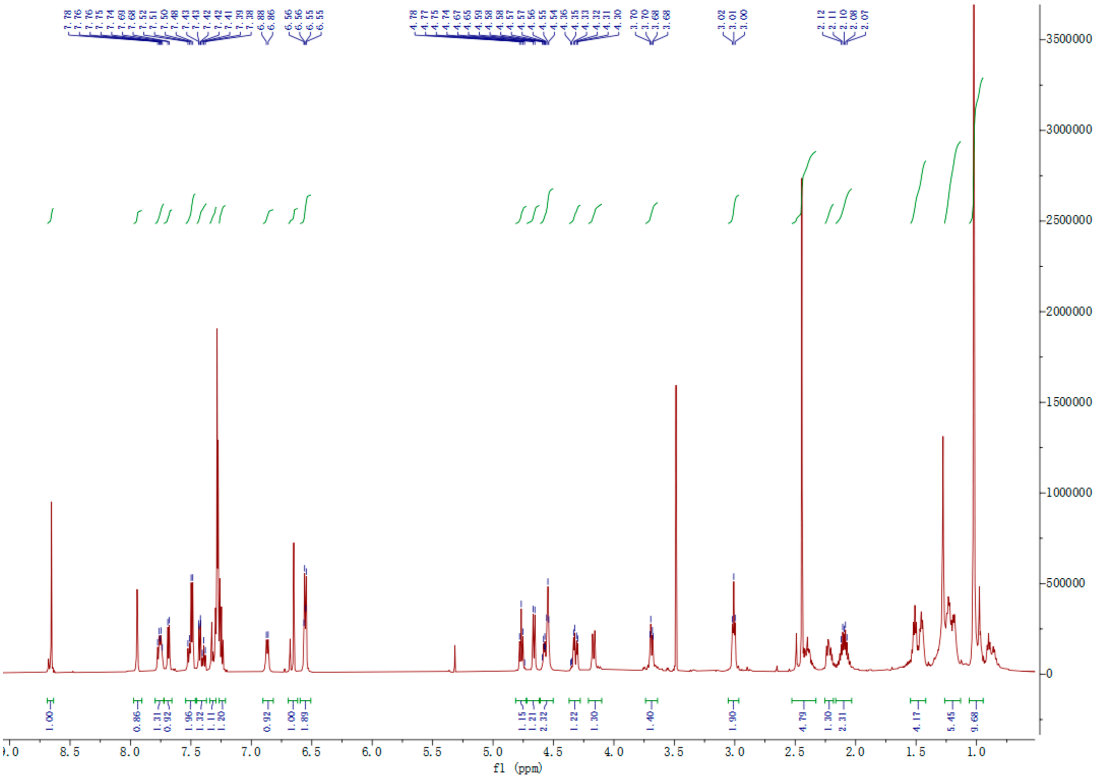

Figure S10. $^{13}$C spectrum of compound **1** (100 MHz, chloroform-d)

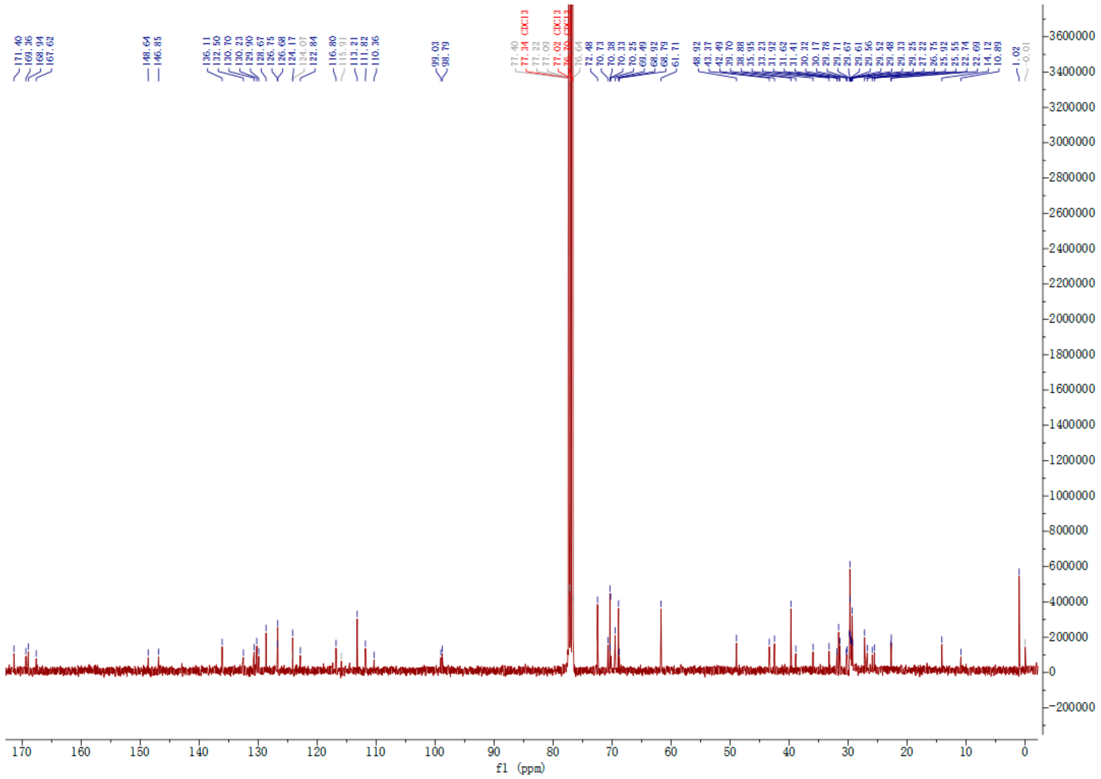


Figure S11. $^{13}$C spectrum of compound **2** (100 MHz, chloroform-d)

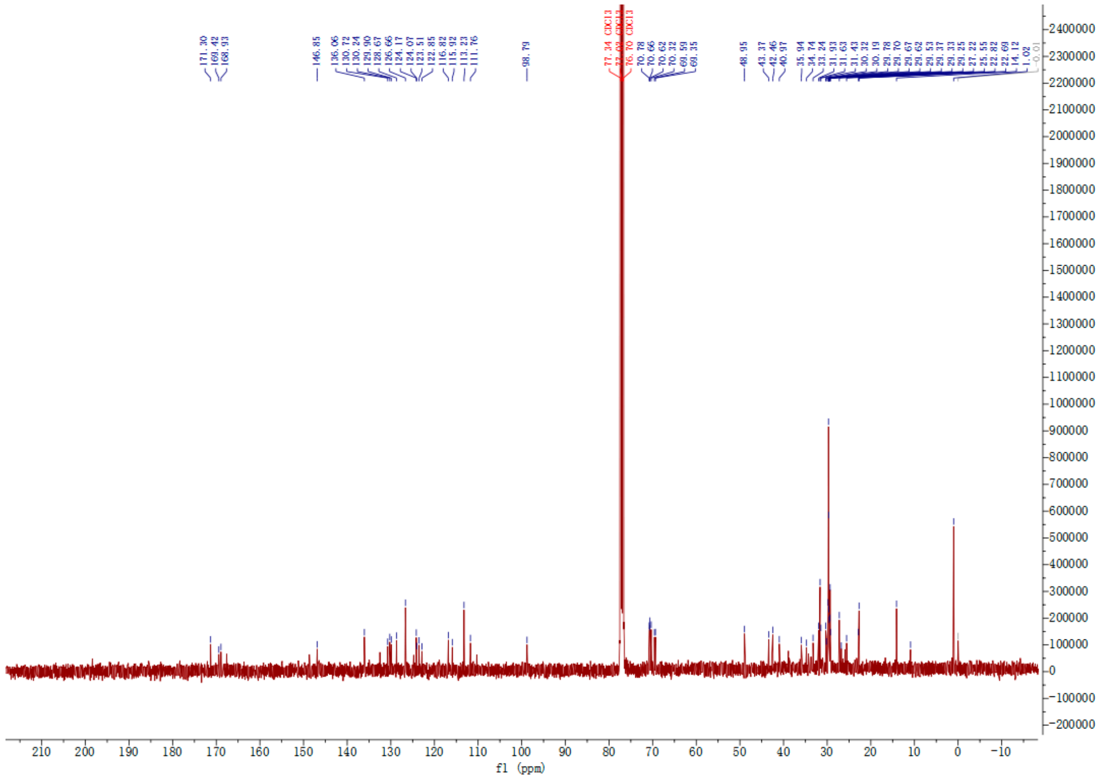

Figure S12. $^{13}$C spectrum of compound **3** (100 MHz, chloroform-d)

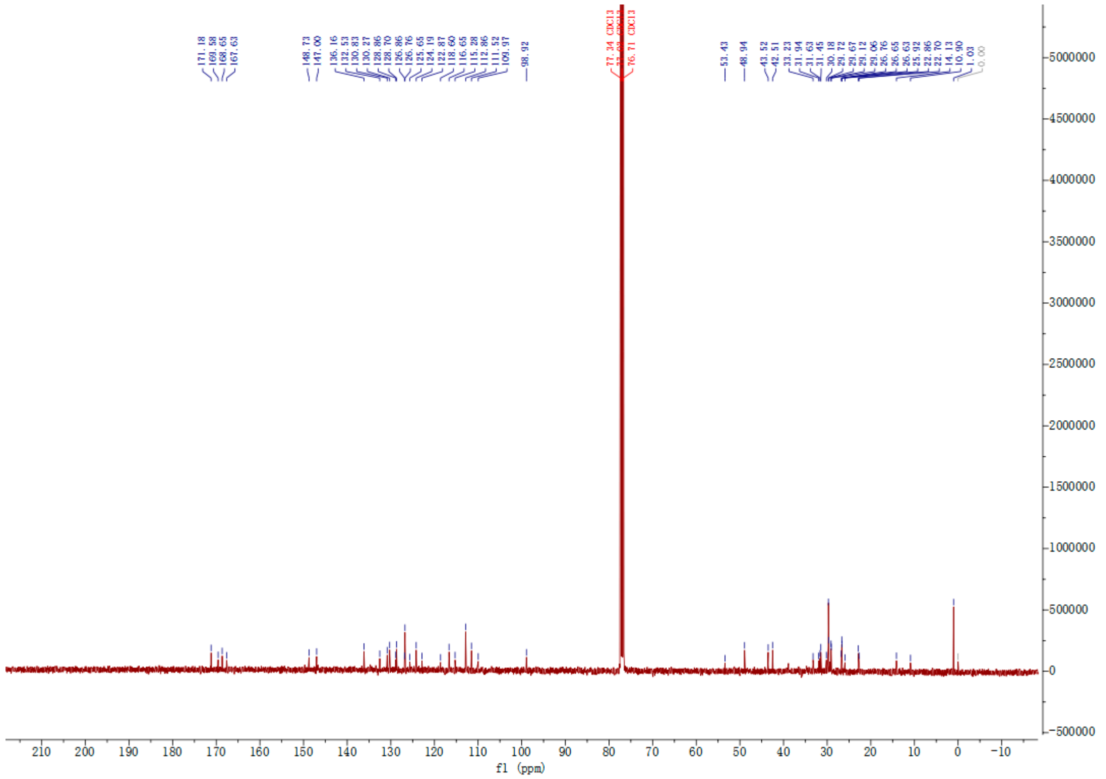

Figure S13. $^{13}$C spectrum of compound **4** (100 MHz, methanol-d4)

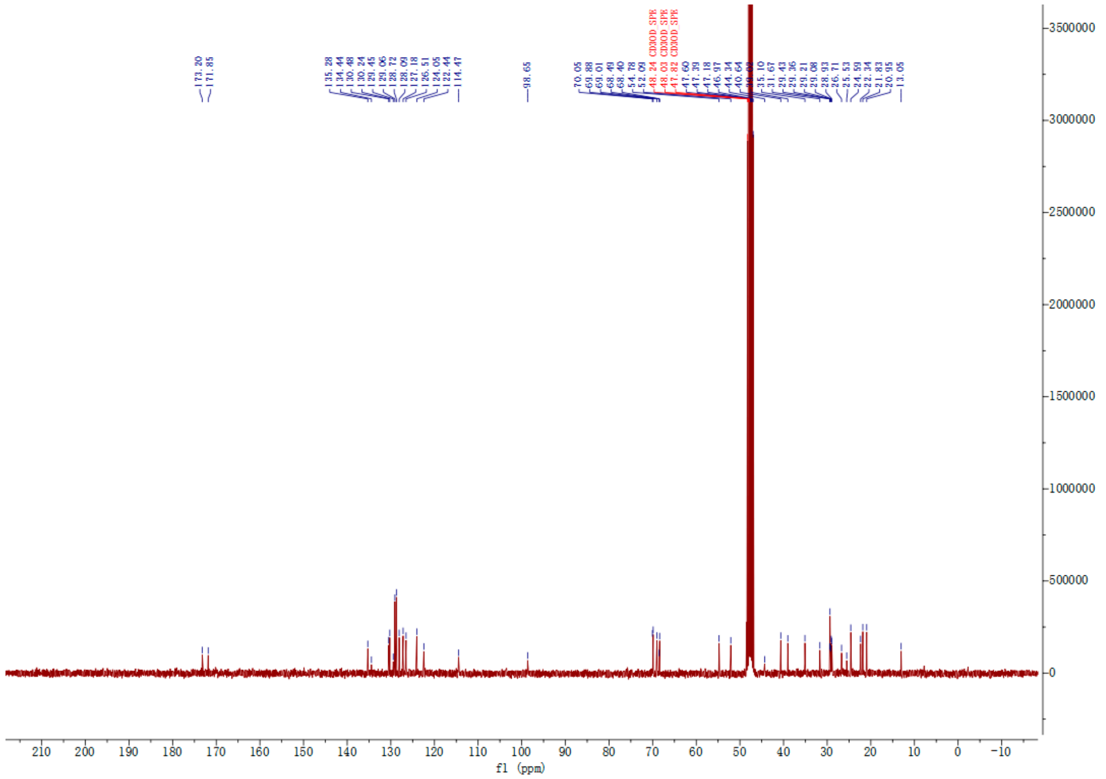

Figure S14. $^{13}$C spectrum of compound **5** (100 MHz, methanol-d4)

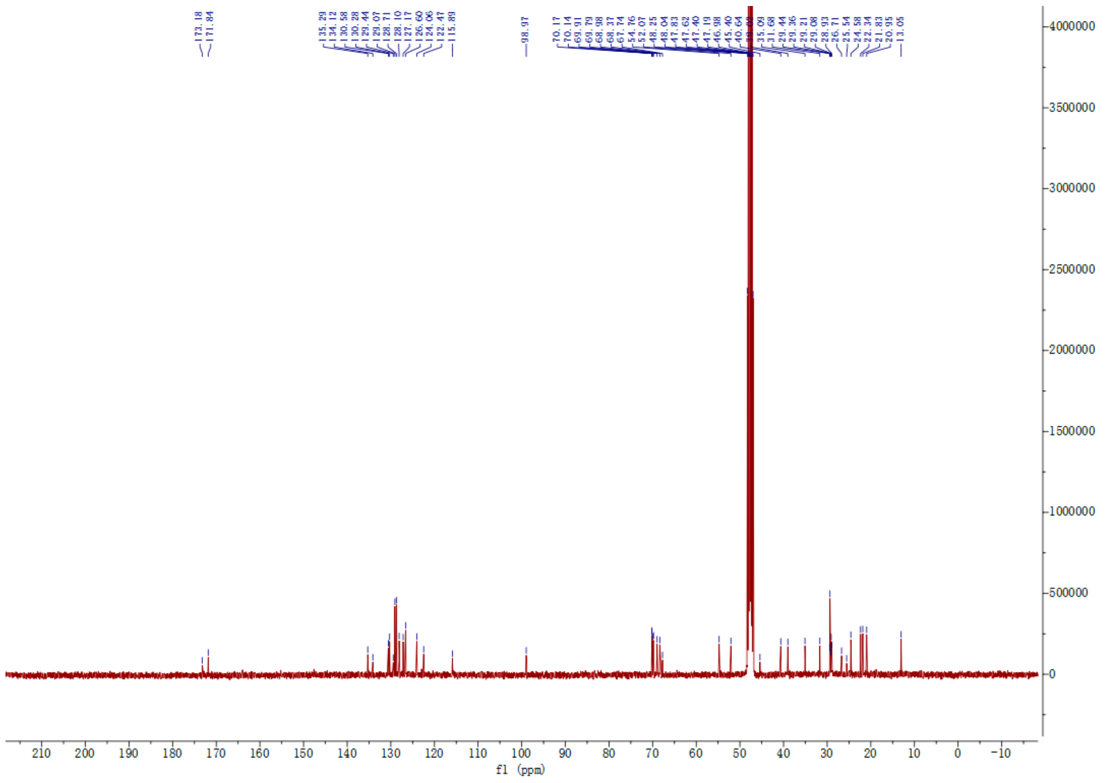

Figure S15. $^{13}$C spectrum of compound **6** (100 MHz, methanol-d4)

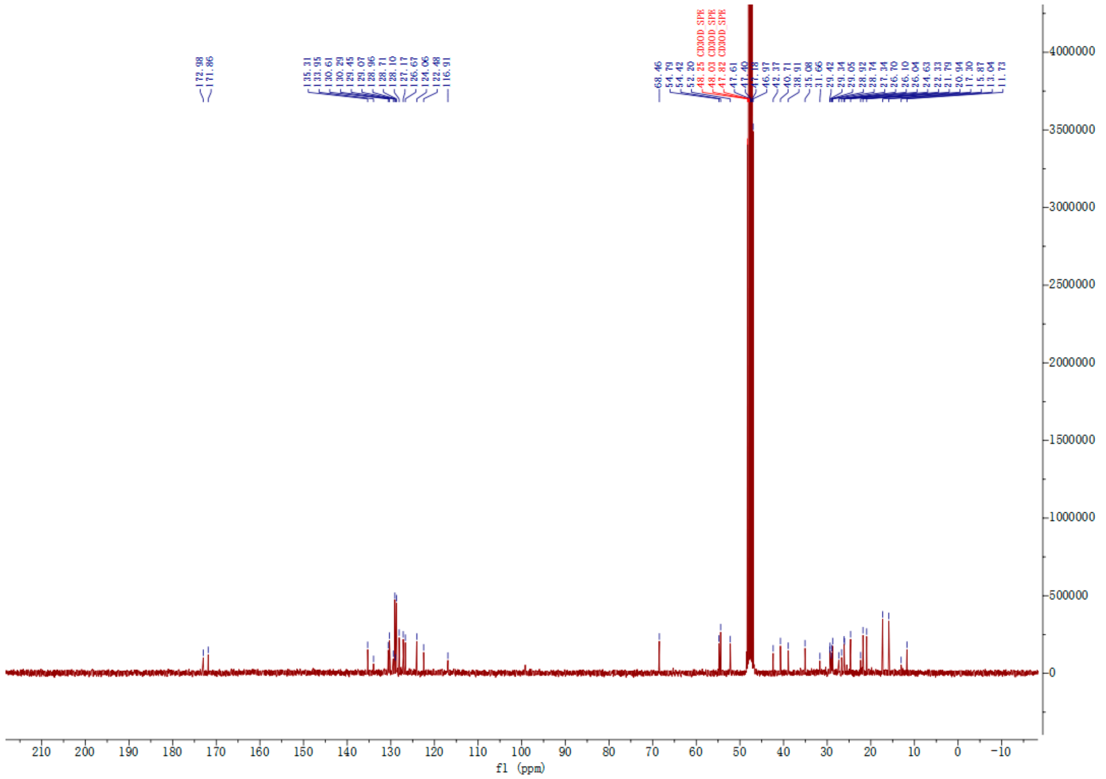

Figure S16. $^{13}$C spectrum of compound **7** (100 MHz, Methanol-d4)

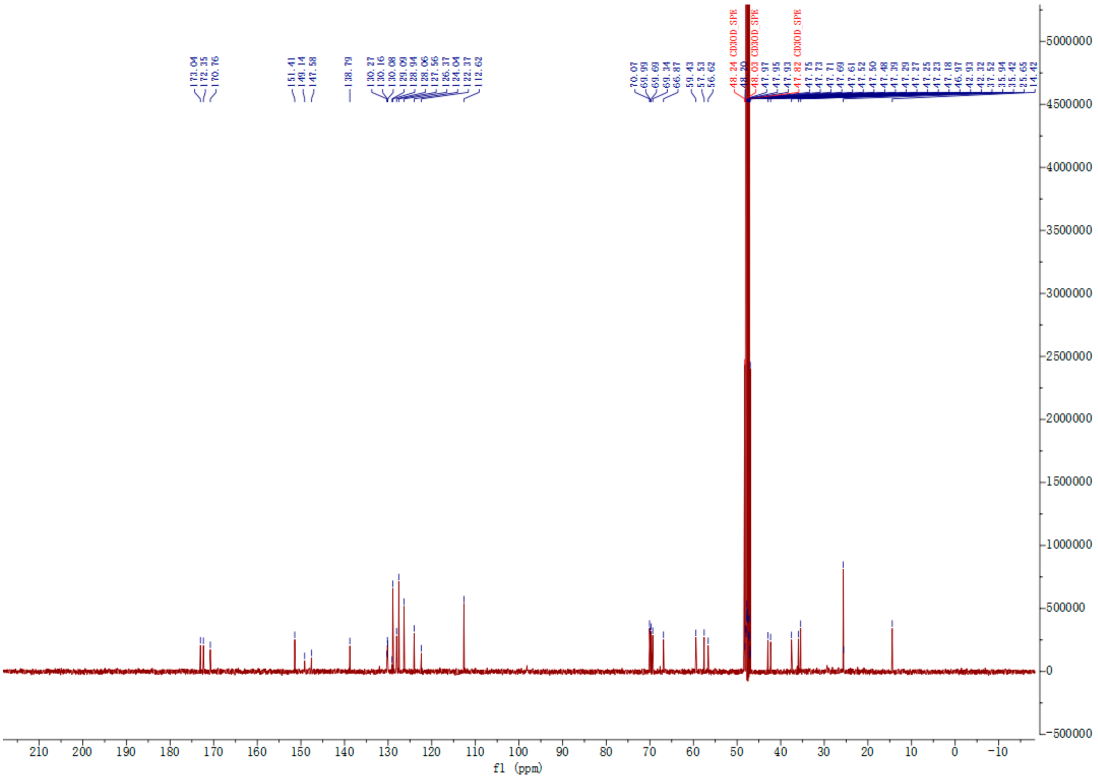

Figure S17. $^{13}$C spectrum of compound **8** (100 MHz, chloroform-d)

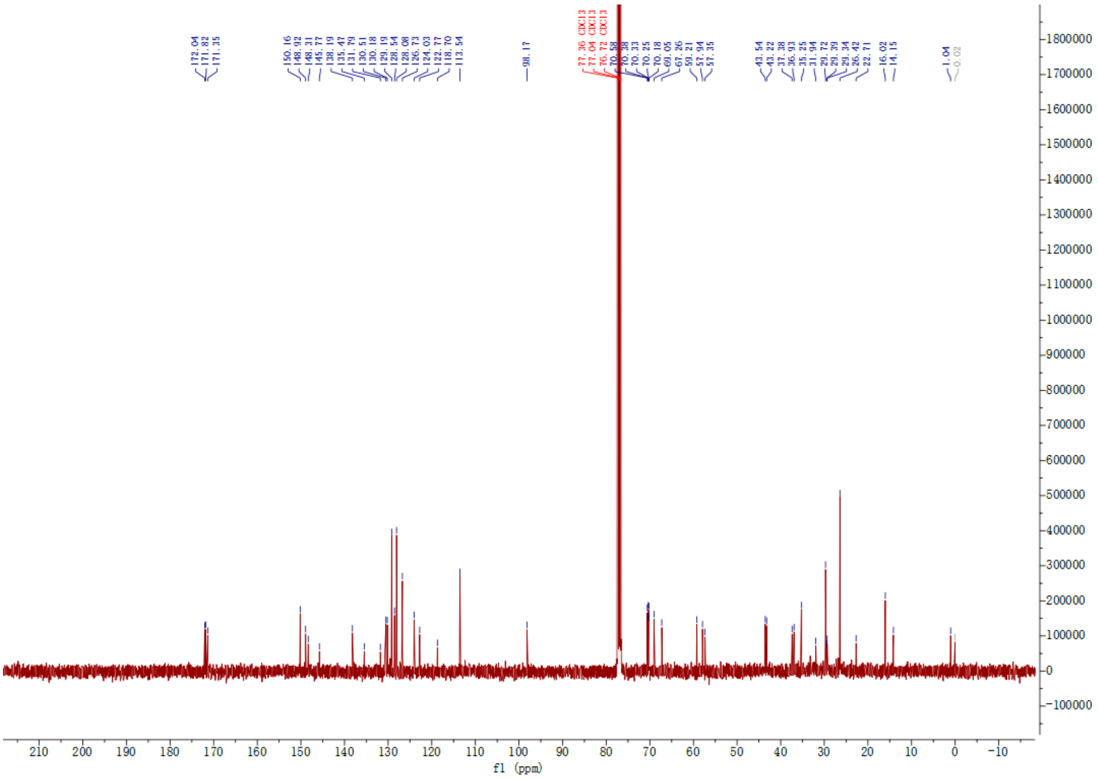

Figure S18. $^{13}$C spectrum of compound **9** (150 MHz, chloroform-d)

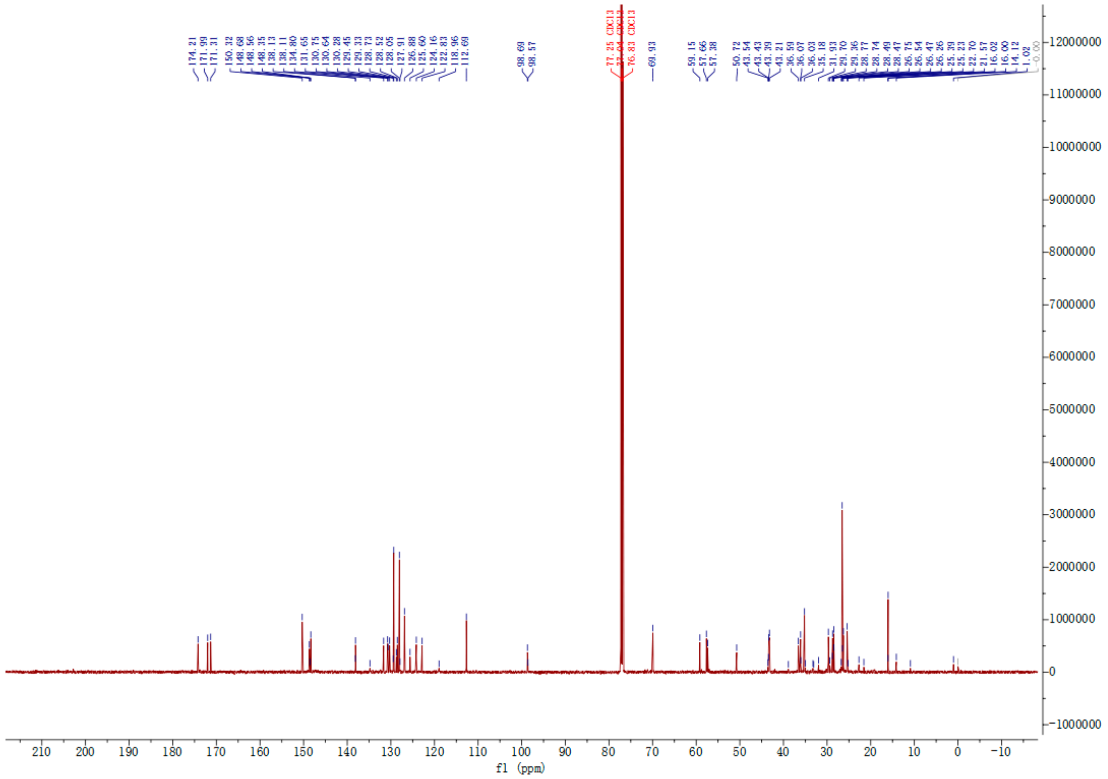

Figure S19. HRMS spectrum of compound **1**

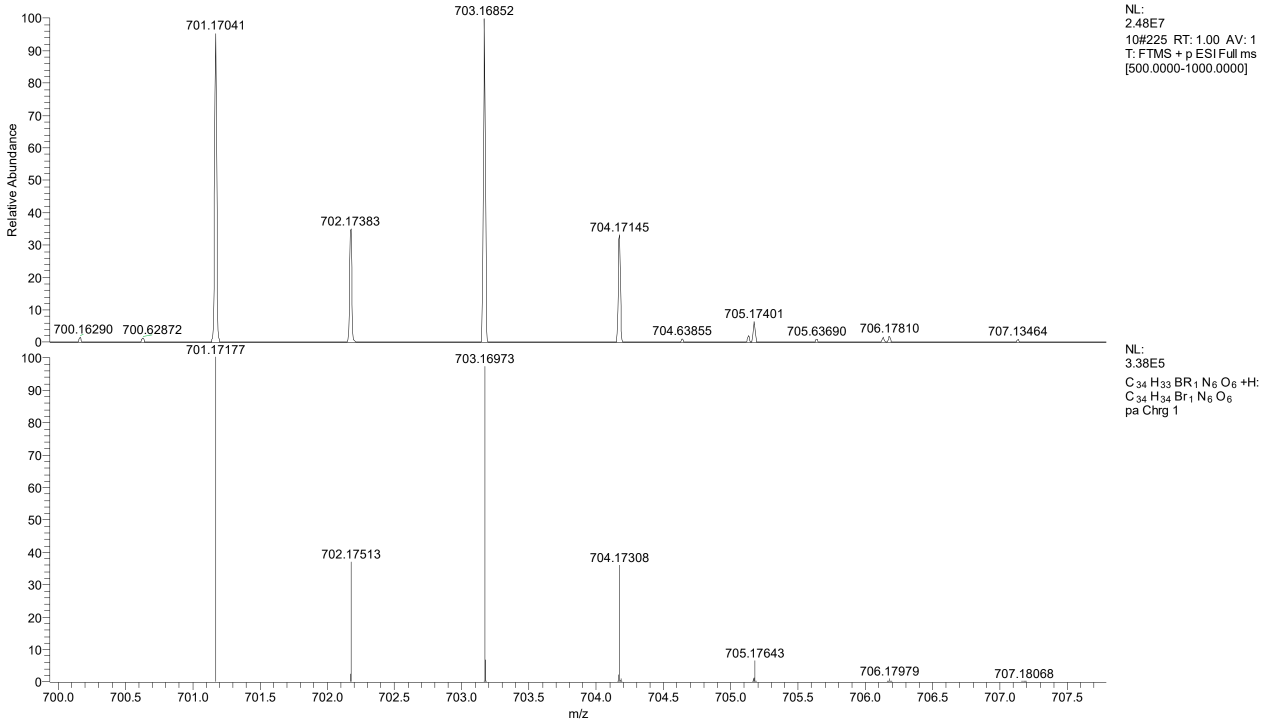


Figure S20. HRMS spectrum of compound **2**

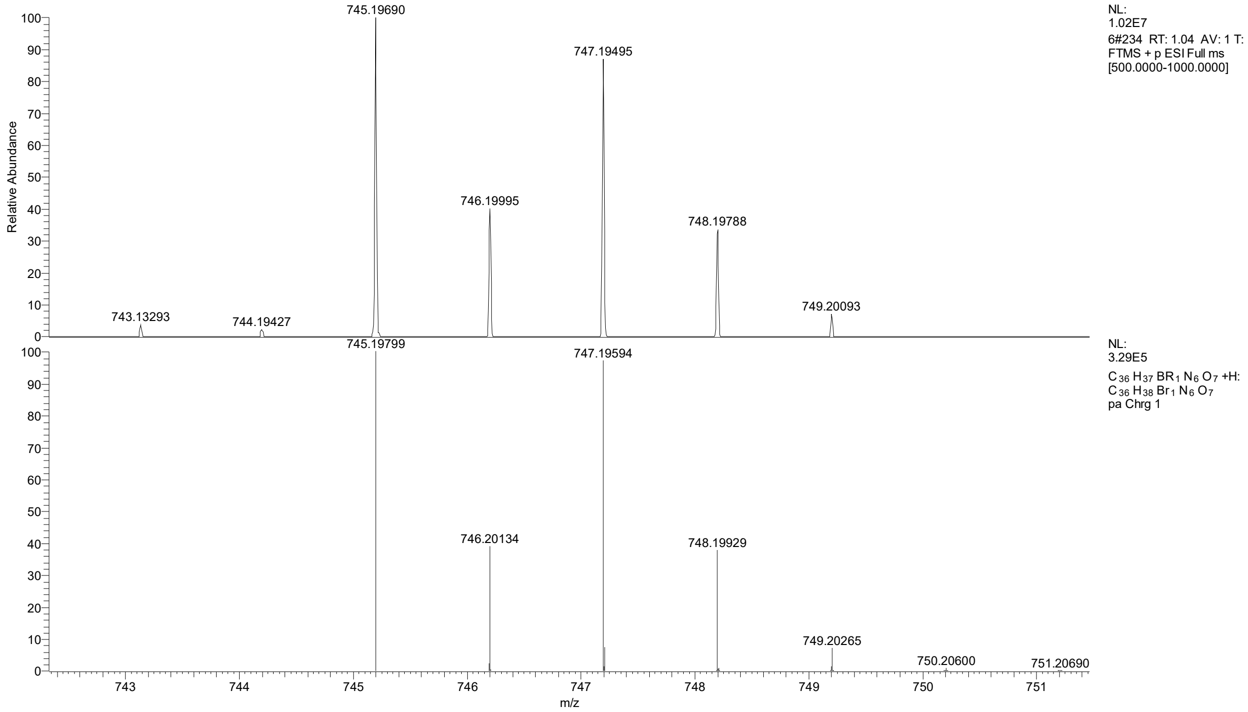

Figure S21. HRMS spectrum of compound **3**

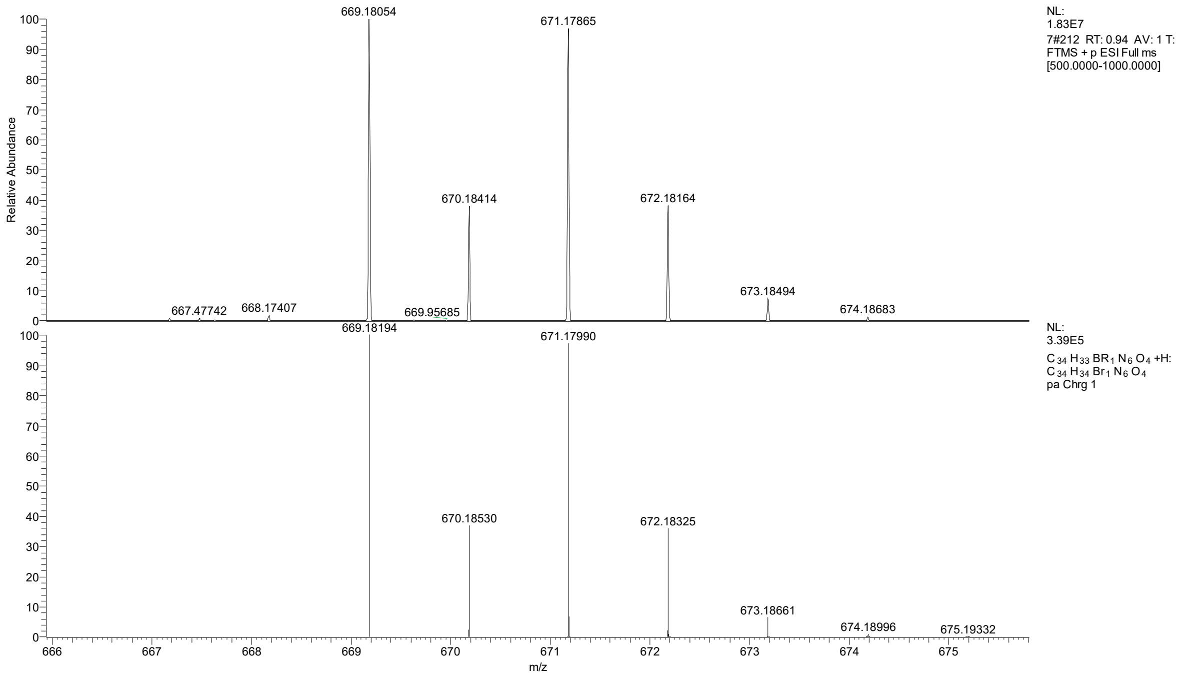


Figure S22. HRMS spectrum of compound **4**

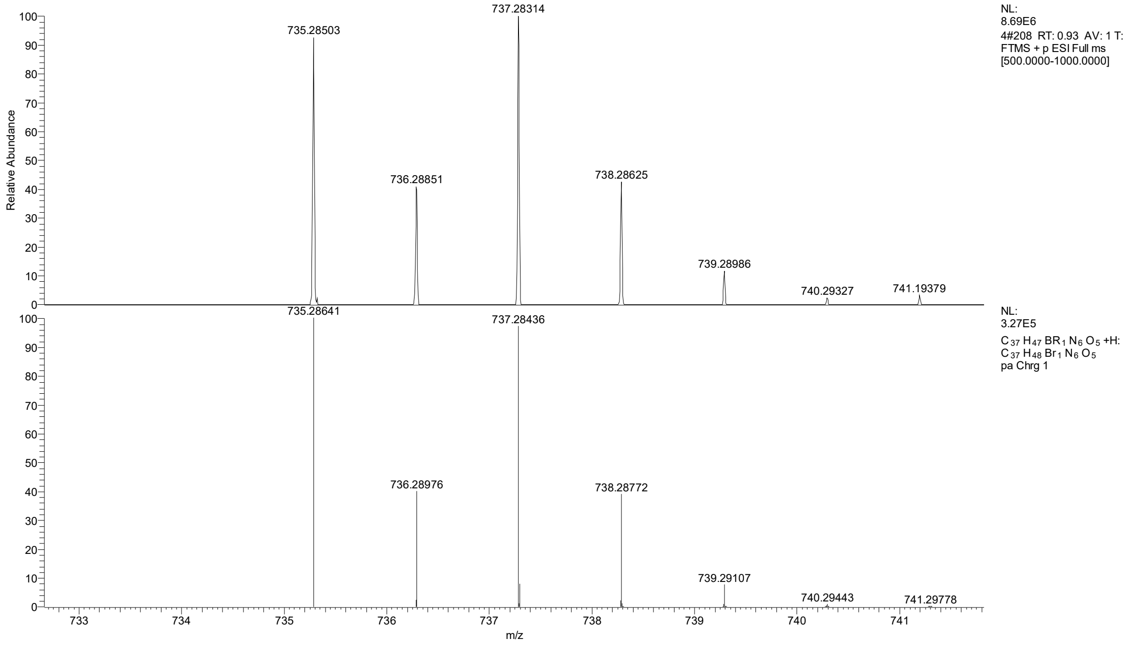

Figure S23. HRMS spectrum of compound **5**

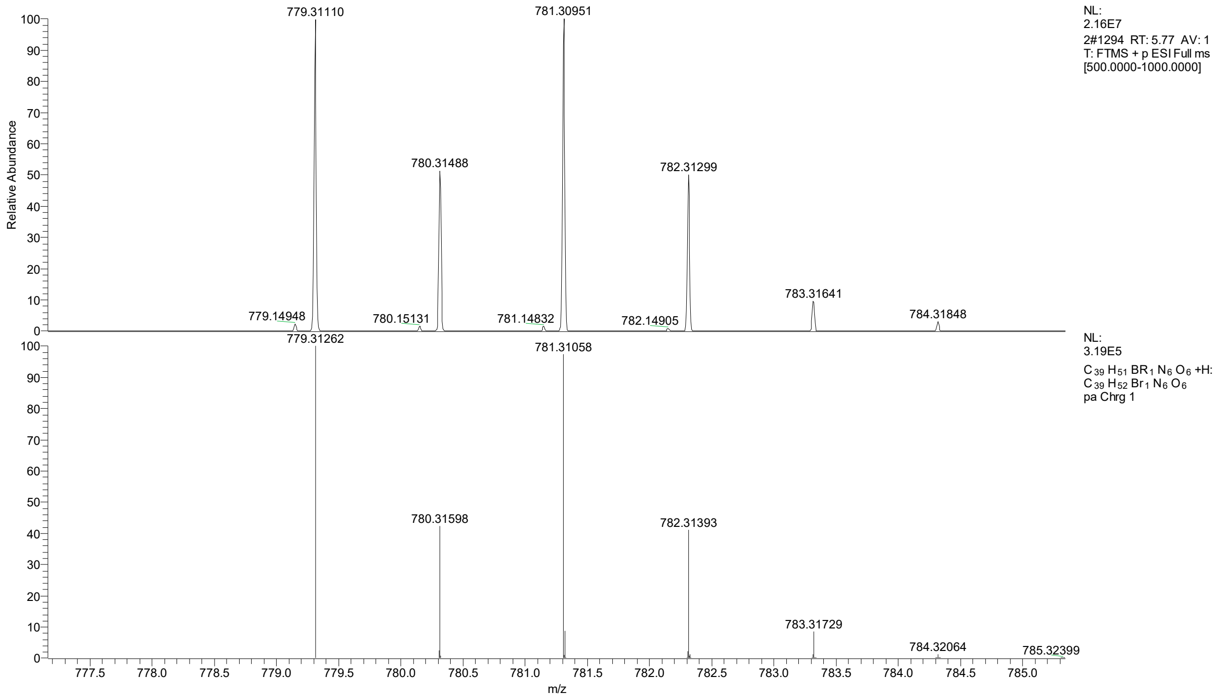


Figure S24. HRMS spectrum of compound **6**

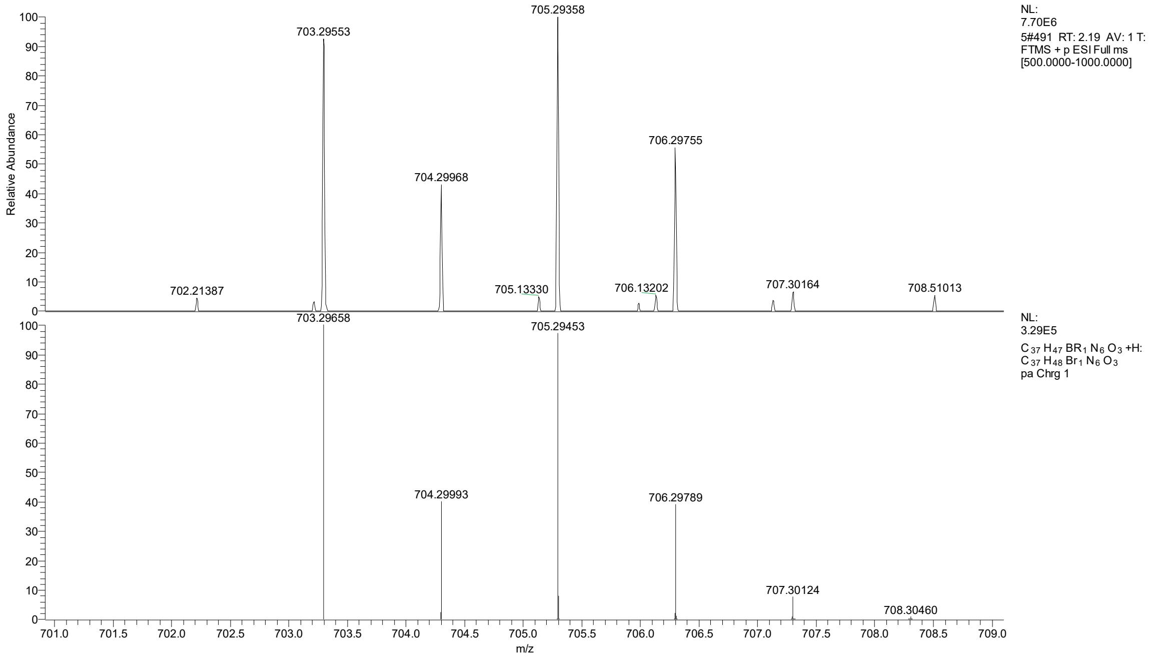

Figure S25. HRMS spectrum of compound **7**

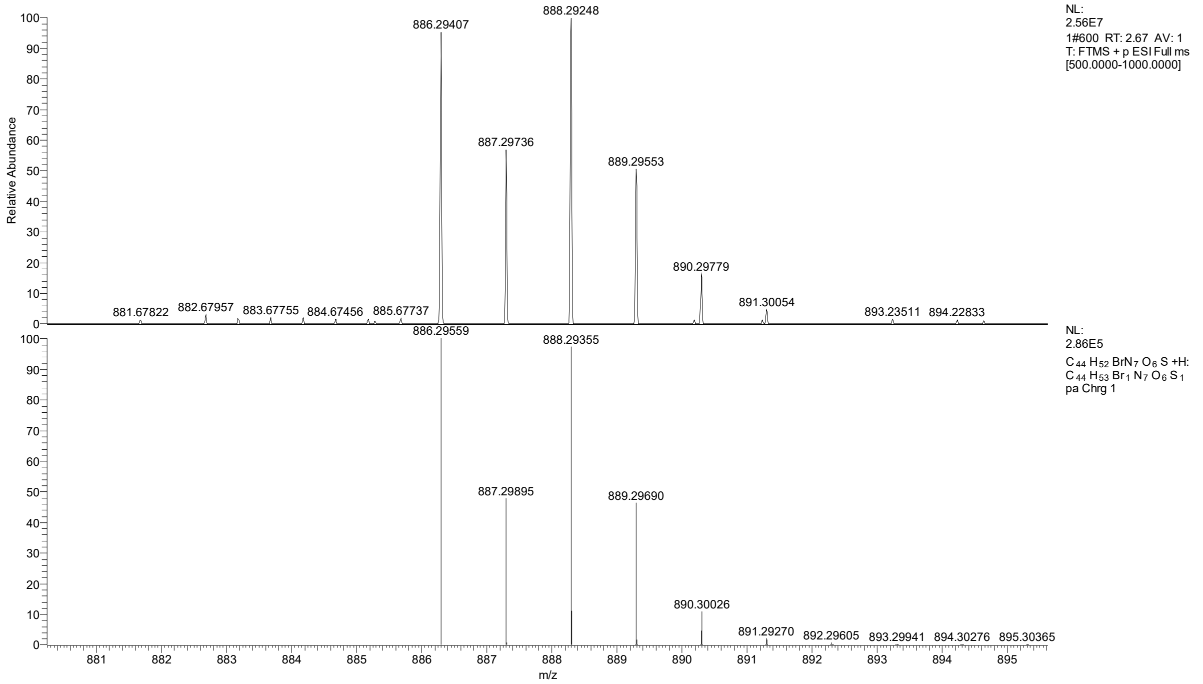


Figure S26. HRMS spectrum of compound **8**

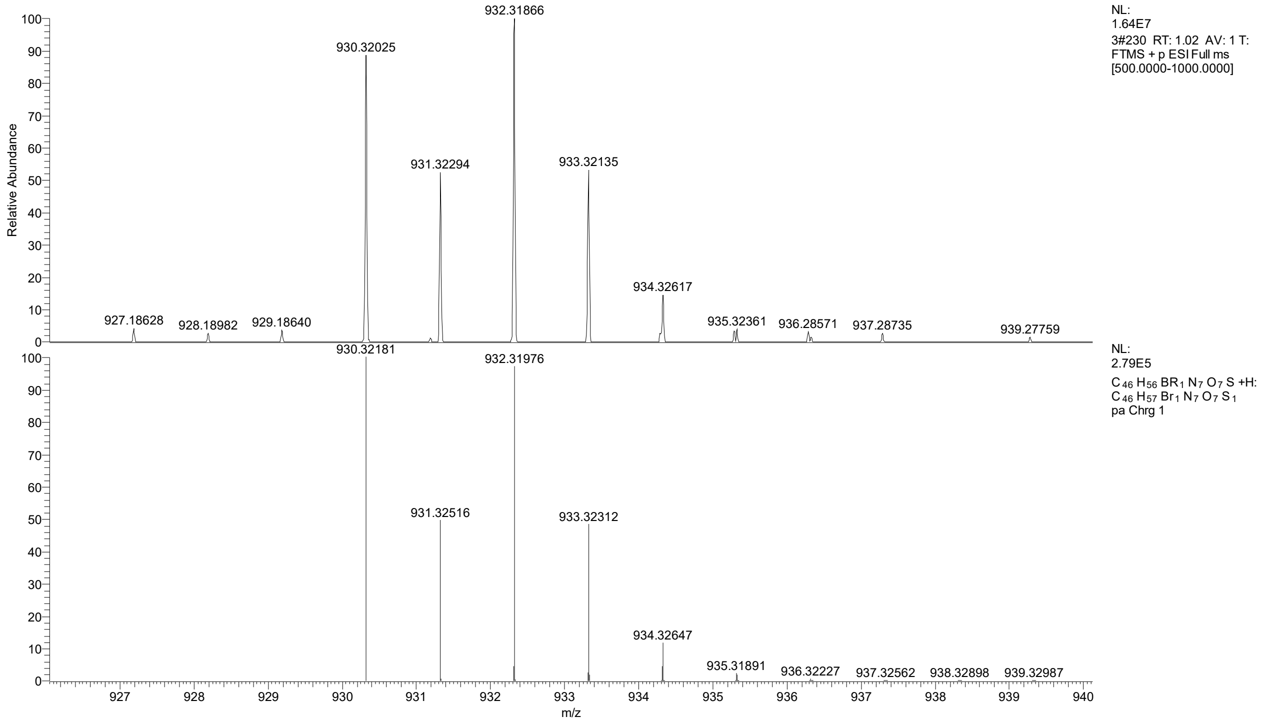

Figure S27. HRMS spectrum of compound **9**

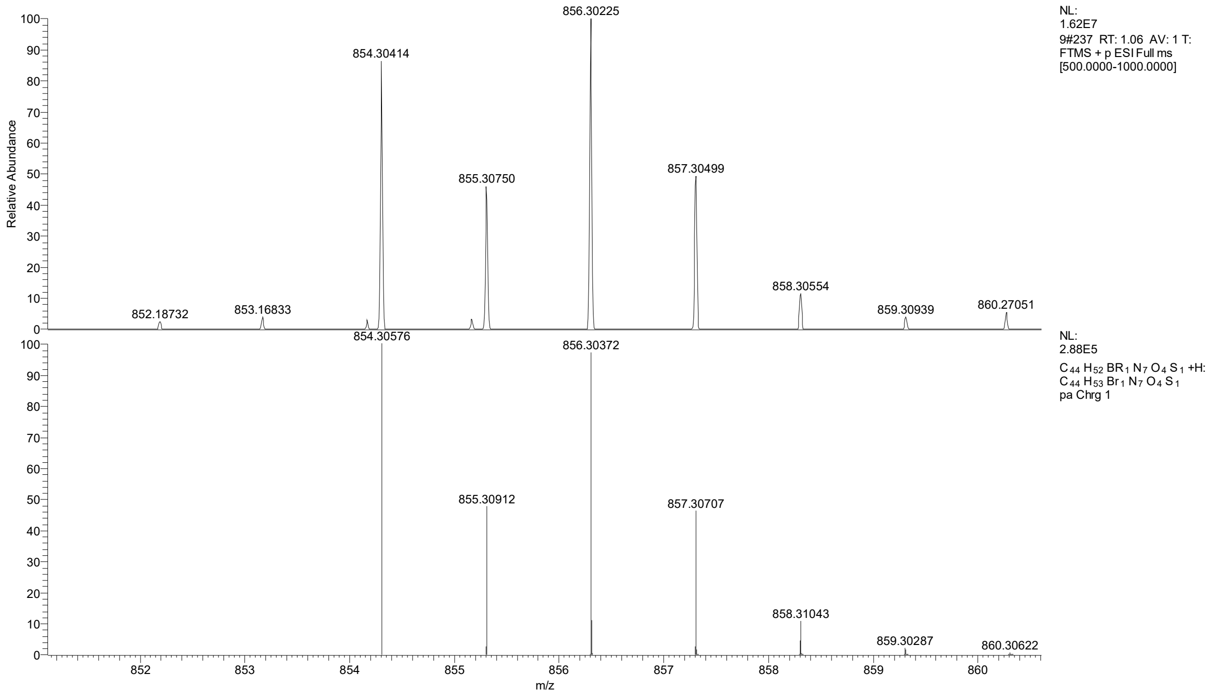

Figure S28. Referential Western Blot image of wild type, SNCA over-expressed, and PFF transfected SNCA over-expressed H293T Cell lines

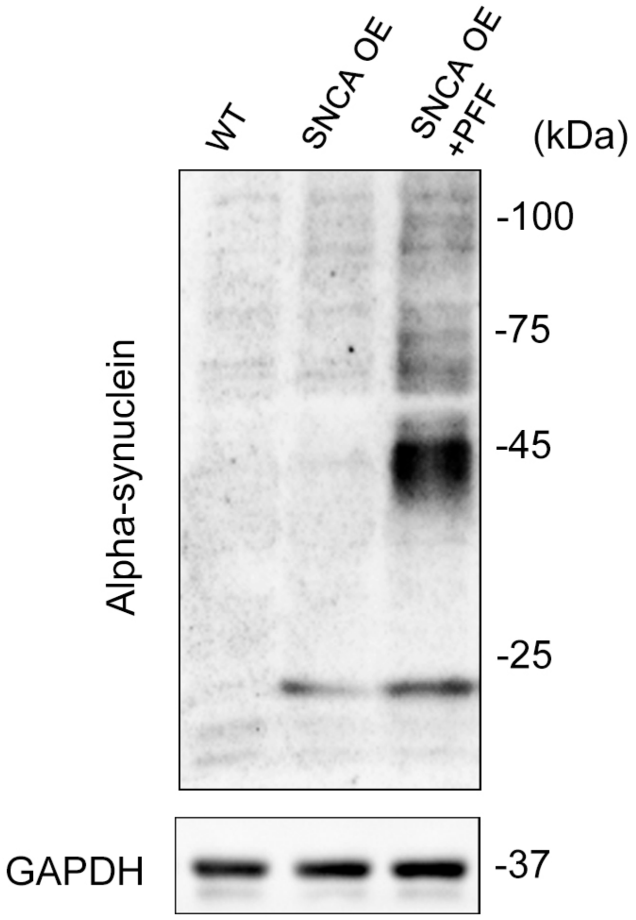

Figure S29. Compounds induced degradation of α-synuclein aggregates

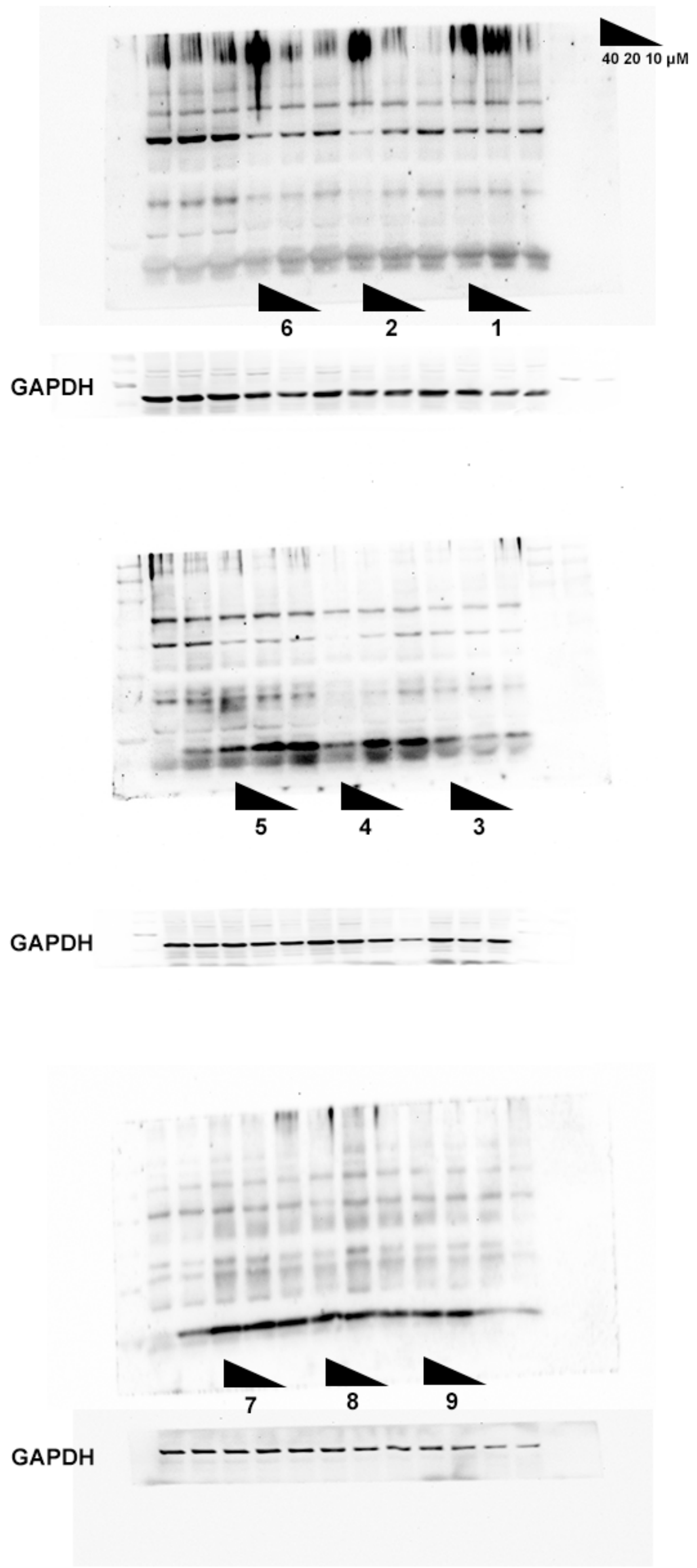

Figure S30. The cell viability data

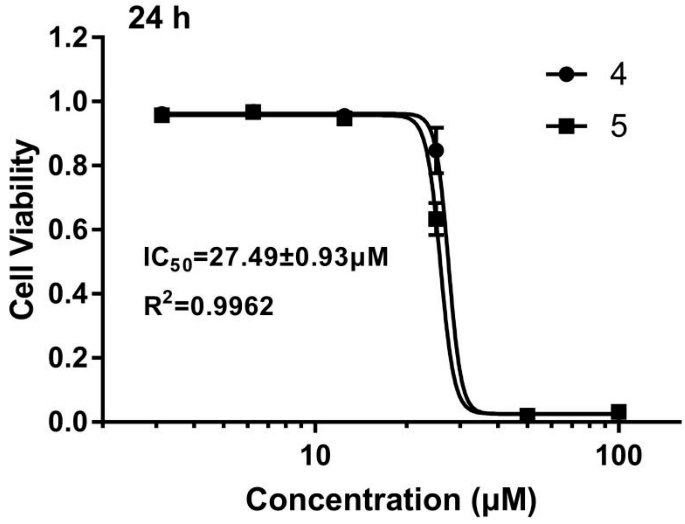


Figure S30. Cytotoxicity assay of H293T cells with two representative compound 4 and 5 (24h). Data reported as mean ± SD of four independent trials of four replicates each. At tested concentrations, compound 4 and 5 showed light toxic effects ($IC_{50}$ = 27.49 ± 0.93 μM) on cell viability.

Table S1. The predicted log*P* values of synthesized compounds

| Compound | Predicted log*P* |
|---|---|
| 1 | 4.43917 |
| 2 | 4.25677 |
| 3 | 6.16229 |
| 4 | 5.49149 |
| 5 | 5.30909 |
| 6 | 7.21462 |
| 7 | 5.95112 |
| 8 | 5.76873 |
| 9 | 7.88763 |

Table S1. The log*P* values of the PROTACs 1-9 were predicted using Chem3D 20.0.0.41.
